# Glycemic Control and the Heart: The Tale of Diabetic Cardiomyopathy Continues

**DOI:** 10.3390/biom12020272

**Published:** 2022-02-08

**Authors:** Miriam Longo, Lorenzo Scappaticcio, Paolo Cirillo, Antonietta Maio, Raffaela Carotenuto, Maria Ida Maiorino, Giuseppe Bellastella, Katherine Esposito

**Affiliations:** 1 Department of Advanced Medical and Surgical Sciences, University of Campania “Luigi Vanvitelli”, 80138 Naples, Italy; miriam.longo@unicampania.it (M.L.); lorenzo828@virgilio.it (L.S.); paolo.cirillo10@hotmail.com (P.C.); antonietta.maio@unicampania.it (A.M.); raffacarotenuto@gmail.com (R.C.); mariaida.maiorino@unicampania.it (M.I.M.); giuseppe.bellastella@unicampania.it (G.B.); 2 Division of Endocrinology and Metabolic Diseases, University of Campania “Luigi Vanvitelli”, 80138 Naples, Italy

**Keywords:** type 2 diabetes, diabetic cardiomyopathy, cardiovascular disease, heart failure, glucose control, glucose-lowering agents

## Abstract

Cardiovascular diseases are the leading cause of death in people with diabetes. Diabetic cardiomyopathy (DC) is an important complication of diabetes and represents a distinct subtype of heart failure that occurs in absence of cardiovascular diseases. Chronic hyperglycemia and hyperinsulinemia along with insulin resistance and inflammatory milieu are the main mechanisms involved in the pathophysiology of DC. Changes in lifestyle favoring healthy dietary patterns and physical activity, combined with more innovative anti-diabetes therapies, are the current treatment strategies to safeguard the cardiovascular system. This review aims at providing an updated comprehensive overview of clinical, pathogenetic, and molecular aspects of DC, with a focus on the effects of anti-hyperglycemic drugs on the prevention of pump dysfunction and consequently on cardiovascular health in type 2 diabetes.

## 1. Introduction

Cardiovascular disease (CVD) represents a challenging issue in the management of diabetes mellitus (DM) and the main cause of death in diabetic patients. Heart failure (HF) is one of the major causes of morbidity and mortality from cardiovascular disease [1]. Recent studies have found that rates of incident HF hospitalization were twofold higher in patients with diabetes compared with those without [1,2]. Diabetic cardiomyopathy (DC) is a subtype of HF defined as the diabetes-associated structural and functional myocardial dysfunction that occurs in absence of conventional cardiovascular diseases (i.e., coronary artery disease, uncontrolled hypertension, valvular heart diseases or congenital heart diseases) [3,4]. DC often appears in the context of CVD; however, it may present as the unique manifestation of cardiac disease [4].

The three major risk factors for DC are high hemoglobin A1c (HbA1c), long disease duration, and older age [4,5]. It has been estimated that 22% of patients diagnosed with idiopathic cardiomyopathy have underlying DM [6]. Moreover, in studies published after 2000, in which more advanced echocardiographic imaging techniques were used, the prevalence of diastolic dysfunction in the population affected by type 2 DM (DMT2) without the conventional CVD varied between 28% and 75%. Data reporting the prevalence rate of DC in type 1 diabetes are limited; however, there is evidence from a few observational studies that type 1 diabetes even affects heart function with different mechanisms as compared with those described in DMT2 [6,7]. 

This review aims at providing an updated comprehensive overview of clinical, pathogenetic, and molecular aspects of DC, with a focus on the effects of anti-hyperglycemic drugs on the prevention of pump dysfunction and consequently on cardiovascular health in DMT2.

## 2. Clinical Aspects of Diabetic Cardiomyopathy

Independently of traditional risk factors, diabetes-related heart anatomical and functional alterations, including fibrosis, left ventricle (LV) remodeling, and impaired contractility, led to the four stages or subgroups of DC (Table 1) [8,9,10]. DC is regarded as a restrictive phenotype with LV hypertrophy and diastolic dysfunction during the first stages, and a dilatative phenotype with reduced ejection fraction at late stages [10]. Specifically, patients with stage 1 are usually asymptomatic and have only diastolic dysfunction. On the other hand, in stages 2 and 3 there are both diastolic and systolic dysfunctions. In patients at early stage, although myocardial dysfunction is subclinical, stress situations (i.e., exercise) can induce an impairment of systolic performance with altered contractile reserve [8,9]. This subclinical systolic dysfunction could be associated with diastolic dysfunction, which precedes the development of both systolic dysfunction with reduced LV ejection fraction (LVEF). Otherwise, patients in stage 4 have symptomatic HF and coronary heart disease [8,9]. Although the distinction of these four forms has important therapeutic and prognostic consequences, its translation in clinical practice is difficult since it is challenging to distinguish when heart abnormalities are due to DC or to coronary artery disease or other diseases unrelated to diabetes [4]. 

### Imaging and Laboratory

Echocardiography, also known as cardiac ultrasound (cUS), is the most sensitive test to diagnose DC [7]. It allows the evaluation of anatomical and functional alterations in the LV, including the diastolic dysfunction, LV filling pressure, and myocardial perfusion reserve [4,7]. Moreover, cUS can also exclude other causes of cardiac damage. The addition of tissue doppler imaging (TDI) currently improves its diagnostic accuracy in measuring myocardial velocities, leading to higher performance in detecting diastolic dysfunction in patients with normal systolic function [7,11]. Cardiac magnetic resonance imaging (cMRI) is the gold standard for the identification of interstitial fibrosis and extracellular matrix expansion, which are important pathological features of patients with DC [7]. The assessment of myocardial fibrosis by cMRI could have a prognostic value in patients with DC without any evidence of underlying CVD, identifying patients at high risk of future cardiovascular events [12]. Interestingly, cardiac abnormalities assessed by cMRI have been associated with the up-regulation of specific microRNAs targeting the extracellular matrix [13].

Among the cardiovascular biomarkers, brain natriuretic peptide (BNP) could be the most useful one to evaluate LV dysfunction, as well as in asymptomatic patients in the subclinical phase [14]. 

Interestingly, persistent microalbuminuria can be used as a marker of diffuse fibrosis and diastolic dysfunction, based on its association with cardiac extracellular volume [15]. 

## 3. Pathophysiology of Diabetic Cardiomyopathy

**Figure 1:**
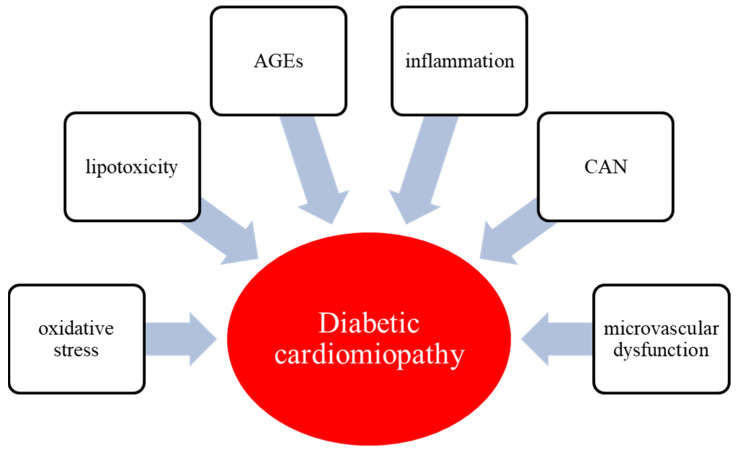
Molecular mechanisms underlying the development and progression of DC. AGEs, advanced glycated end-products; CAN, cardiac autonomic neuropathy.

A wide variety of molecular mechanisms underlie the development and progression of DC, which are partially different from those promoted by hypertension or ischemia (Figure 1). Hyperglycemia and hyperinsulinemia along with insulin resistance are the main pathogenetic drivers of the metabolic alterations that lead to the DC [4,7]. In the setting of hyperglycemia, insulin resistance, and hypertriglyceridemia, there is a reduction in the myocardium’s ability to use glucose as an energy source, and it subsequently switches to free fatty acids (FFAs). Specifically, the key mechanisms involved in the pathophysiology of DC are oxidative stress, hyperglycemia and glucotoxicity, lipotoxicity, advanced glycated end-products (AGEs) deposition, inflammation, cardiac autonomic neuropathy (CAN), and microvascular dysfunction [16]. 

### 3.1. Hyperglycemia and Glucotoxicity 

Hyperglycemia is a major etiological factor in the development of DC. It enhances the production of FFA and different growth factors, causing abnormalities in substrate supply and utilization, calcium homeostasis, and lipid metabolism [17]. Furthermore, it contributes to the excessive release of reactive oxygen species (ROS), which are responsible for oxidative stress, impairment of gene expression, abnormal signal transduction, and cardiomyocytes apoptosis. The effects of growth factors on connective tissue, together with fibrosis and the formation of AGEs, increase the stiffness of diabetic hearts [18]. A healthy heart can metabolize a range of substrates, including FFA, glucose, amino acids, ketones, and lactate, in order to produce ATP [19,20]. Particularly, the oxidation of FFA, glucose, and pyruvate represent the main contributors for ATP formation and energy storage. The activity of pyruvate dehydrogenase (PDH), which is a key enzyme regulating the balance between carbohydrate and fat metabolism in the heart, appears to be decreased in diabetes and associated with the impairment of the pyruvate oxidation [21,22]. The dissociation of glycolysis and pyruvate oxidation in the diabetic heart results in the accumulation of glycolytic intermediates, which are responsible for the activation of specific glucose-sensing transcription factors [23,24]. Glycolytic intermediates decrease the sarcoplasmic reticulum calcium ATPase 2a (SERCA2a) expression [25], which is essential in calcium homeostasis, resulting in diastolic dysfunction [26]. Indeed, SERCA2a expression in the heart appeared to be decreased in diabetes [26,27]. Finally, hyperglycemia induces the production of AGEs and decreases the production of nitric oxide (NO) in coronary endothelial cells, leading to upregulation of endothelial vascular growth factor (VEGF) pathway. This metabolic abnormality causes fibrosis of capillaries with consequent impairment of myocardial functional reserve [7]. Therefore, uncontrolled hyperglycemia and subsequent metabolic changes triggered by these factors are responsible for a status of cardiac glucotoxicity and myocardial dysfunction [28].

### 3.2. Lipid in Diabetic Cardiomyopathy

The relationship between dyslipidemia, inflammation, and cardiovascular (CV) health in type 2 diabetes mellitus is complex. The status of insulin resistance and chronic low-grade inflammation increases plasma triglycerides (TG) levels [29]. Inflammatory mediators also enhance the production of matrix-metalloproteinases (MMPs) that, together with TG, reduce high-density lipoprotein cholesterol (HDL) levels. All these factors promote structural and functional impairment of the CV system [30]. Increased circulating TG levels are associated with intracardiac accumulation of fatty acids and, thus, enhanced fatty acid β-oxidation and impaired insulin metabolic signaling in diabetic hearts [31]. Additionally, in diabetes, cardiomyocytes often display increased expression of peroxisome proliferator-activated receptor-α (PPAR-α), associated with increased FFA uptake, triacylglycerol accumulation, and reduced glucose utilization [32]. Abnormal lipid metabolism often accelerates the development of DC. Indeed, there is an increased release of FFA from adipose tissue, which in turn leads to the FFA transport in myocyte sarcolemma [33]. The cluster of differentiation 36 (CD36), which is located predominantly on the cell surface, promoting the FFA uptake in both sarcolemma and endosomal membranes, is overexpressed in diabetic hearts [34]. Conversely, GLUT4, which is an important glucose membrane transporter, is internalized in the cardiomyocytes, assuming an intracellular location [34]. The reduced glucose uptake promotes a metabolic shift toward FFA oxidation, resulting in decreased cardiac performances [35,36]. Indeed, CD36 knockout mice showed a 70% reduction in FFA uptake in cardiomyocytes [37], and CD36 deficiency prevents lipotoxic cardiomyopathy [38,39]. Some lipid metabolites, including diacylglycerols and ceramides, impair insulin metabolic signaling, further promoting cardiac damage. Increased levels of diacylglycerols in cardiomyocytes compromise glucose metabolism through activation of protein kinase C (PKC) isoforms, thereby reducing insulin metabolic signaling and NO production [31]. Therefore, excessive accumulation of lipids and toxic intermediates contributes to cardiac insulin resistance, reduced NO bioavailability, inflammation, fibrosis, and diastolic dysfunction in DC [32].

### 3.3. Oxidative Stress

In the setting of diabetes, both increased production of ROS and impairment of endogenous antioxidant mechanisms play an essential role in both mitochondrial damage and cardiac fibrosis [40]. In DC, the antioxidant factors, like superoxide dismutase (SOD) and glutathione peroxidase (GSH-Px), are reduced in cardiomyocytes, and ROS generation is dramatically increased, leading to high oxidative stress [41]. Moreover, the continuous production of ROS induces the activation of the TGFβ1/Smad3 signaling pathway, which promotes the expression of several fibrotic factors, as α-SMA, collagen I, and collagen III in diabetic heart [42]. Therefore, therapeutic molecules and agents that counteract ROS generation and the related cardiac fibrosis may represent a potential therapeutic strategy for DC.

### 3.4. Endothelial Dysfunction

The integrity of vascular endothelial cells constitutes the essential key basis of CV health. A healthy endothelium generates various vasodilating factors, such as NO, prostacyclin, bradykinin, and endothelium-derived hyperpolarizing factor; moreover, it also produces vasoconstrictor agents including endothelin and angiotensin II. Hyperglycemia can impair the physiological homeostasis of the endothelium. This may result in increased leukocyte adhesion and permeability, and reduced fibrinolysis, activating the diacylglycerol (DAG)-PKC signaling pathway [43,44]. PKC is a family of serine-threonine kinases that has been previously reported to be associated with vascular permeability in diabetes [45,46]. Activation of this pathway may contribute to diabetic endothelial dysfunction in several tissues, including the heart, retina, and kidney [47]. However, the precise pathway that leads to increased endothelial cell permeability and leukocyte adhesion by PKC activation remains still unclear.

### 3.5. Inflammation

Inflammation has been recognized as a key common pathogenic process of lipid and glucose excess. Since DM is recognized as an inflammatory disease, the induction of pro-inflammatory genes and proteins has been reported as one of the main risk factors for myocardial injury [16]. In addition, an inflammatory myocardial injury may be amplified by increased ROS levels. The nuclear factor-KB (NF-kB) is mainly implicated in inflammatory responses. NF-KB induces the expression of proinflammatory cytokines, such as TNF-α, IL-6, pro IL1-β, and pro-IL-18 in the heart. NF-kB can also induce the expression of NLRP3 inflammasome [48]. The latter seems to be a novel molecular marker of DC. The activation of NLRP3 inflammasome leads to recruitment of procaspase-1. Activated caspase-1 processes many inflammatory mediators, such as interleukin-1β and interleukin-18 precursors. An NF-κB–positive feedback further increases NLRP3 inflammasome assembly and procaspase-1 activation and pro-interleukin-1β processing and maturation, generating an inflammatory vicious cycle [49]. Meanwhile, increased monocyte/macrophage migration through the coronary endothelium increases resident cardiac macrophages, which acquire a pro-inflammatory profile (M1) under conditions of enhanced oxidative stress induced by increased ROS and reduced NO bioavailability [50]. All these pathways contribute to cardiomyocytes dysfunction and the development of DC.

### 3.6. AGEs

There is evidence that AGEs contribute to the development and progression of diabetic heart dysfunction. Chronic hyperglycemia results in intra- and extracellular AGEs production, by non-enzymatic reactions between reducing sugars with amino groups of nucleic acids, lipids, peptides, and proteins. Once formed, AGEs interact with receptors for advanced glycation end products (RAGEs), triggering a series of vascular and myocardial damage, resulting in diastolic and systolic dysfunction [51]. AGEs may bind RAGE, leading to maladaptive inflammatory gene expression, thus activating mitogen-activated protein kinase (MAPK) and Janus kinase (JAK) pathways in vascular and cardiac tissues. Meanwhile, AGEs are involved in increasing the production of ROS, which further promotes inflammation and fibrosis [33]. AGEs-induced modification of extracellular matrix and other structural proteins may provide a reduction of myocardial compliance and myocardial fibrosis [16]. Interestingly, in a mouse model of type 1 diabetes, administration of a RAGE antagonist prevented AGEs/RAGE signaling-mediated increase in myocardial collagen, fibrosis, stiffness, and diastolic dysfunction [52].

### 3.7. CAN

Hyperglycemia can activate multiple pathways involved in the pathogenesis of CAN. The majority of these pathways are related to the metabolic and/or oxidative state of neuronal cells. Oxidative stress can induce DNA damage, leading to activation of PARP and inhibition of GAPDH [53]. This in turn can activate multiple molecular mechanisms, including the polyol pathway, the hexosamine pathway, as well as activation of PKC and increased production of AGEs, which correlate with the severity of autonomic nerve abnormalities in patients with DM [53,54]. Particularly, the hyperadrenergic state characterizing the CAN promotes the development of interstitial fibrosis and diastolic dysfunction, also through the increased activation of the renin–angiotensin–aldosterone system (RAAS) [55].

## 4. Role of Glucose Control in Diabetic Cardiomyopathy

There is evidence that poor glycemic control correlates with an increased risk of heart failure among individuals with diabetes [56]. About a 1% increase in HbA1c is associated with an 8% increased risk of heart failure [56,57]. In a recent Chinese study on 64 adult subjects with type DMT2 and 30 healthy controls, LV myocardial function, evaluated by cMRI, was impaired in patients with uncontrolled DMT2 (HbA1c ≥ 7%) and diabetes duration < 10 years as compared with healthy control [58]. Moreover, poor glucose control was an independent predictor of LV myocardial dysfunction in this population. Furthermore, high HbA1c variability is associated with increased risk of all-cause and cardiovascular mortality, as well as diabetic complications [59]. These findings suggest that achieving adequate glycemic control may be useful to reduce the burden of diabetes-related CVD and thus preserve CV health in people with diabetes. In a recent meta-analysis with meta-regression of 15 cardiovascular outcome trials (CVOTs) including 138,250 patients with T2DM, there was a robust relationship between the reduction in achieved HbA1c at the end of the trial and the HR reduction for major cardiovascular events, mainly driven by the reduction of non-fatal stroke, with no effect on HF [2].

A multifactorial approach that includes lifestyle modification and appropriate medical treatment of DMT2, hypertension, and dyslipidemia is essential to preserve pump function in people with diabetes. Several different new classes of glucose-lowering agents have been recently introduced in the market. Some of them have shown many cardioprotective effects beyond their ability to control hyperglycemia, with beneficial effects in preventing cardiovascular mortality and hospitalization due to HF (Table 2). However, with the exception of some experimental preclinical researches and very limited clinical studies, there is little evidence on the effects of glucose-lowering treatment in the specific setting of DC. 

## 5. Lifestyle Changes

A healthy dietary pattern together with regular physical activity, which represent the first line therapeutic strategies for T2DM, may be useful to prevent and treat DC in individuals with diabetes [33]. Aerobic exercise and caloric restriction of fat and refined carbohydrate intake seem to improve insulin resistance, reduce myocardial triglyceride content, and improve diastolic LV dysfunction [33]. Indeed, in a study of 11 overweight-to-obese male patients with T2DM, 12-weeks of progressive endurance/strength training was effective in improving insulin sensitivity, LVEF, cardiac index, and cardiac output, with no variation in cardiac lipid content [60]. A Mediterranean dietary pattern characterized by a high proportion of mono and polyunsaturated fat, a small portion of red meat, and high quantities of olive oil has demonstrated many cardiovascular benefits, by decreasing inflammatory status, and improving lipid profile, endothelial dysfunction, and insulin sensitivity [61,62]. In particular, in a recent observational study on 9316 men and 27,645 women, including also people with diabetes, a higher adherence to a Mediterranean-style diet evaluated by modified Mediterranean Diet Scores (mMDS) was associated with a 12% reduced risk of HF in men but not in women [63]. Consequently, a healthy lifestyle, including the assumption of the Mediterranean diet, can be useful to preserve heart pump function in T2DM.

## 6. Diabetes Therapy and DC

Nowadays, there are several treatment options targeting differing mechanisms of actions for improving glycemia, in people with DM and CVD. In the following paragraph, we will discuss the effects of anti-hyperglycemic agents and lipid-lowering therapy on CV health in DM.

### 6.1. Metformin

Metformin is the first-line oral anti-hyperglycemic treatment in the management of DMT2. It belongs to the class of biguanide drugs. Initially, its use was contraindicated in patients with diabetes and HF, due to the potential increased risk of lactic acidosis. In 2006, the FDA removed this contraindication from metformin’s product label, and a further systematic review showed that metformin was associated with reduced all-cause mortality, without increased risk of hospital admission in patients with HF and diabetes [64]. 

However, metformin should be stopped in case of acutely decompensated HF, sepsis, or hypoperfusion conditions in order to prevent lactic acidosis. Metformin seems to exert several favorable effects on the pathophysiology of DC, by decreasing insulin resistance and TNF-α production, reducing cardiomyocytes and fibroblast LV remodeling, increasing the production of NO, and improving systolic and diastolic function [7,65]. 

Both in vivo and in vitro studies showed that metformin can improve cardiac function and alleviate apoptosis by activating the prokineticin 2 pathway [7]. Moreover, in murine models, metformin provided substantial protection against DC, reducing the production of ROS, expression of p53, and production of collagen [66].

In a clinical setting, in a retrospective cohort study on 242 adults with DMT2 undergoing coronary angiography, the use of metformin was associated with improved LV diastolic function (lower IVRT and higher e’ wave), as compared with sulfonylurea or insulin therapy [67]. Moreover, a 2-years prospective, randomized, open-label trial on 49 non-diabetic patients with metabolic syndrome and diastolic dysfunction showed that treatment with metformin (1000 mg bid) on top of lifestyle counseling was associated with significant improvement of diastolic function (improved e’ velocity and reduced insulin resistance) as compared with lifestyle counseling alone [68]. A meta-analysis of 13 trials conducted between 1995 and 2011 with a total of 2.079 individuals with DMT2 demonstrated that the use of metformin slightly improved the risk of all-cause mortality, cardiovascular death, myocardial infarction, and peripheral disease as compared with control strategies (no intervention, placebo, or lifestyle changes), but none of these outcomes achieved statistical significance [69]. 

### 6.2. Sulfonylureas

Sulfonylureas are a class of oral anti-diabetes agents that stimulate the release of insulin from pancreatic β cells. In experimental murine models, triple anti-diabetes oral therapy with glimepiride, metformin, and rosiglitazone showed a synergistic effect in improving insulin resistance and retarding development of DC [70]. However, there is evidence that associates the use of sulfonylureas with a higher risk of hypoglycemia and weight gain [71], which in turn increases the risk of HF. Moreover, in a cohort study on new users of metformin or sulfonylurea in the veteran’s population, the initiation of sulfonylurea was associated with a 32% increased risk of HF hospitalization and cardiovascular death compared with metformin initiation [72]. In a pooled analysis of clinical trials, the use of sulfonylureas did not significantly increase the risk of MACE, cardiovascular death, or HF hospitalization [73]. At the moment, sulfonylureas are not indicated as first-line treatment for patients with DMT2 and cardiovascular disease by international guidelines for the management of DMT2 [71]; therefore, their use should be considered in people free of CVD in case of intolerance to metformin or when an upgrade of glucose-lowering therapy is needed.

### 6.3. Thiazolidinediones 

Thiazolidinediones are oral anti-hyperglycemic agents that act on peroxisome proliferator-activated receptor-γ (PPR-γ) to increase insulin sensitivity in peripheral tissues. Both clinical and experimental studies suggest beneficial effects of these molecules on inflammation, lipid and protein metabolism, and vascular endothelial function [74,75]. 

In preclinical studies on diabetic mice, the use of pioglitazone remarkably increased ventricular function, reduced fibrosis and TGF-β protein expression in myocardial tissues, and attenuated myocardial hypertrophy [75]. Moreover, pioglitazone combined with curcumin reduced oxidative stress and fibrosis in diabetic rats, decreasing lipid peroxidation and TGF-β1 level [76]. However, the use of these drugs in clinical practice is limited due to their potential to cause weight gain, edema, fractures, and HF. Indeed, the addition of pioglitazone and rosiglitazone in clinical trials demonstrated a significant increase in the risk for heart failure hospitalizations [77,78]. Therefore, these agents should be avoided in patients with HF and used with caution in patients who have any signs or symptoms of HF.

### 6.4. SGLT-2 Inhibitors

Sodium-glucose cotransporter 2 inhibitors (SGLT-2i) are the latest class of oral glucose-lowering agents approved by FDA for management of DMT2. These drugs inhibit the renal sodium/glucose cotransporter in the kidney proximal convoluted tubule, increasing glycosuria and favoring osmotic diuresis [79].

Over the last few years, there has been a growing body of evidence from randomized controlled trials of their cardiovascular and renal benefits in people with DMT2 at high cardiovascular risk [80]. Although it is noticeable that SGLT-2i exert beneficial cardiovascular effects, the specific mechanisms of action of SGLT-2i on cardiac metabolism have not yet been carefully understood. The potential mechanisms involved in the cardiovascular protection are the glucose-lowering effect, weight loss, blood pressure reduction, hemodynamic effects, osmotic diuresis, and natriuresis. Conversely, SGLT-2 seems to not be expressed in cardiac myocytes [81]. However, SGLT-2i may have a direct action on the myocardium by the inhibition of the sodium-hydrogen exchanger (NHE), which may be involved in myocardial injury, fibrosis, and LV dysfunction [82]. 

In preclinical studies, dapagliflozin was demonstrated to reduce ROS production, decrease fibrosis, reduce inflammation, and improve systolic function in an experimental model of DC [83]. When added to ticagrelor, dapagliflozin ameliorated the progression of DC, attenuating the activation of NLRP3 inflammasome, and reducing TNFα and IL-6 levels and fibrosis [84]. The use of empagliflozin in diabetic mice for 8 weeks was associated with decreased myocardial oxidative stress, reduced myocardial fibrosis, and better myocardial structure and function [85]. Moreover, empagliflozin improved diastolic function and reduced mortality in a murine model of severe diabetes [86].

In a prospective observational study of 35 symptomatic non-ischemic HF patients with DMT2 and LVEF > 40% treated with empagliflozin (EMPA group) and 20 control subjects, patients in the empagliflozin group showed a significant improvement of LV dysfunction [LV global longitudinal strain (LVGLS) and the ratio of early diastolic mitral inflow velocity to early diastolic mitral annular velocity (E/e′)], particularly in early cardiomyopathy stages [87]. Data from a meta-analysis of the CVOTs involving the use of SGLT2-i versus placebo show a significant 33% reduced risk of hospitalization for HF in people using this class of drugs [73,88]. Since this effect is associated with each studied molecule (dapagliflozin, empagliflozin, canagliflozin, ertugliflozin, and sotagliflozin), the reduced risk for HF related to SGLT-2i should be considered a class effect [79]. Indeed, according to the Standards of Medical Care in Diabetes 2021 of the American Diabetes Association, a SGLT-2i with proven cardiovascular benefit is recommended as part of the glucose-lowering regimen, independent of HbA1c, for patients with type 2 diabetes who have an established history of HF, and particularly with reduced ejection fraction (HFrEF, LVEF < 45%) [71]. Thus, the use of SGLT2i should be recommended as soon as possible in patients with type 2 diabetes and HF at early stages. 

### 6.5. GLP-1 Receptor Agonists

Glucagon-like peptide-1 receptor agonists (GLP-1RAs) are a class of injective anti-diabetes agents that lower blood glucose levels, enhancing insulin secretion in response to nutrient ingestion, without risk of hypoglycemia. Moreover, they inhibit inappropriate glucagon secretion, suppress appetite, and slow gastric emptying, which represent all pleiotropic favorable effects in the management of DMT2 [89].

Over the years, GLP-1RAs have demonstrated many beneficial effects on the cardiovascular system. These agents may exert their benefits both indirectly by decreasing different established cardiovascular risk factors (hyperglycemia, obesity, high blood pressure, and lipid profile) and directly on cardiomyocytes and coronary vasculature [90]. The expression of GLP-1 receptor has been reported in human atrial tissue, vascular smooth muscles, and endothelial cells [91]. GLP-1RA seem to reduce inflammatory myocardial remodeling and decrease inflammatory pathways in cardiomyocytes, promoting glucose uptake and increasing coronary blood flow [90]. 

Exenatide, a short-acting glucagon-like peptide-1 (GLP-1) receptor agonist, has proven to attenuate ROS production, increasing the antioxidant enzymes manganese-dependent superoxide dismutase (MnSOD) and catalase activity and preventing cell apoptosis [92]. Treatment of lean mice with liraglutide showed to increase myocardial glucose oxidation, enhancing pyruvate dehydrogenase activity and alleviating diastolic dysfunction in mice with DMT2 [93]. In a preclinical study on streptozotocin-induced diabetic rats treated with liraglutide and/or empagliflozin, both agents were effective in ameliorating myocardial fibrosis and apoptosis. Particularly, empagliflozin modulated fatty acid and glucose metabolism, while liraglutide regulated inflammation and apoptosis in DC [94].

Whether GLP-1RAs are effective in improving ventricular function in human subjects is still controversial. A 5-week continuous subcutaneous infusion of GLP-1 (2.5 pmol/kg/min) in 12 patients with New York Heart Association (NYHA) class III–IV HF significantly improved LV function, walking time, and quality of life scores in patients with severe HF [95]. However, the exciting results of the earlier pilot study were not completely confirmed in larger studies of GLP-1RA in patients with HF. Among 154 recently hospitalized patients with HFrEF, the use of liraglutide did not lead to greater post-hospitalization clinical stability for HF as compared with placebo in a 6-months randomized controlled clinical trial [96]. On the other hand, in another randomized controlled trial of 49 subjects with DMT2 without cardiovascular disease assigned to liraglutide (1.8 mg once daily) or placebo, treatment with liraglutide for 6 months reduced early LV diastolic filling and LV filling pressure, thereby improving diastolic function [97].

In the randomized, double-blinded, placebo-controlled multicenter LIVE trial, 241 subjects with reduced LV ejection fraction (LVEF ≤45%) with and without diabetes were randomized to liraglutide or placebo [98]. As a result, liraglutide did not improve LV systolic function as compared with placebo in stable chronic heart failure patients and was rather associated with an increase in heart rate and more serious cardiac adverse events. However, a recent meta-analysis of the eight CVOTs with GLP-1RAs so far conducted, including 60,080 participants, demonstrated that GLP-1RAs reduced the risk of MACE by 14%, the risk of cardiovascular death by 13%, and the risk of hospitalization for HF by 10% [99]. 

Although this class of agents has been associated with potential benefits on the risk of HF, further studies are needed to specifically assess the efficacy of GLP-1RAs in the setting of DC. 

### 6.6. DPP-4 Inhibitors

Dipeptidyl peptidase-4 inhibitor (DPP-4i) represents another new class of oral glucose-lowering agents that block the enzyme DPP-4, thereby extending the lifetime of incretin hormones [GLP-1 and glucose-dependent insulinotropic polypeptide (GIP)] and enhancing insulin secretion in a glucose-dependent manner. Pre-clinical studies reported a correlation between DPP-4 activity and cardiac dysfunction [100], as well as the improvement of pump function with the use of DPP-4i [101]. 

In experimental murine models, sitagliptin showed to down-regulate the JAK/STAT signaling pathway—ameliorating histological cardiac structure, oxidative stress, and inflammation—and rejuvenated the antioxidant defenses in diabetic hearts [102]. Sitagliptin could also improve the contraction and relaxation functions of cardiomyocytes in diabetic rats [103].

In a small 24-weeks randomized trial, the use of sitagliptin in subjects with T2DM inadequately controlled with metformin and glyburide was associated with improvement in diastolic function as compared with NPH insulin [104]. Furthermore, an improvement of diastolic function reflected by attenuation of the increase in E/e′ ratio was also observed with sitagliptin compared with placebo in a 24- months, randomized trial of 115 Japanese subjects with DMT2 whose blood glucose was inadequately controlled with lifestyle interventions and/or pharmacotherapy [105]. On the contrary, the use of sitagliptin at the dosage of 50 mg once daily did not produce a significant effect on diastolic function in 80 subjects with DMT2 compared with voglibose (0.6 mg once daily) in a 24-weeks randomized trial [106]. Despite these potential beneficial effects, data from the larger CVOTs with DPP-4i have resulted in a substantial non-inferiority versus placebo with respect to the risk of MACE. Moreover, some concerns have been raised on the possibility of a higher risk of HF hospitalization combined with the use of some molecules in specific trials. Indeed, in the SAVOR-TIMI trial, treatment with saxagliptin was associated with a 7% increased risk of HF hospitalization as compared with placebo [107]. A similar, although not statistically significant trend has been detected also in the EXAMINE trial with the use of alogliptin [108] and CAROLINA trial with linagliptin [109], whereas no difference was observed in the TECOS trial with sitagliptin [110]. 

However, in a recent umbrella meta-analysis, the use of DPP-4i was not associated with a significant increase in HF risk [73]. Thus, this effect seems to be drug-specific and imputable to the sole use of saxagliptin. 

### 6.7. Lipid-Lowering Therapy

Drugs that lower plasma lipids seem to improve cardiac performance in diabetic rats. Statins are effective, pleiotropic lipid-lowering agents representing the first-line therapy for patients with diabetes and dyslipidemia and a cornerstone of prevention of CVD [111]. In previous animal experiments, statins were hypothesized to prevent DC by multiple pleiotropic properties, including anti-inflammatory and antioxidant effects [111]. Rosuvastatin reduced NLRP3 inflammasome and IL-1β activation by suppressing MAPK pathways in diabetic rats [112]. Simvastatin also exhibited protective effects by significantly reducing cardiac enzymes, inflammatory mediators, and deposition of collagen in the diabetic murine heart [113]. On the other hand, an 8-weeks study on 87 murine models with type 1 diabetes rather suggested that glycemic control was more important than pravastatin to attenuate the progression of DC, expressed as the cardiac expression of collagen I/III, matrix metalloproteinase (MMP)-2, MMP-9, and angiotensin-converting enzyme (ACE) [114]. Atorvastatin was able to preserve the myocardial structure, inhibiting fibrosis through anti-apoptosis and anti-inflammation pathways [115]. Atorvastatin could also reduce β-adrenergic dysfunction in rats with diabetic cardiomyopathy via increasing NO availability [116]. Recently, the combined administration of metformin and atorvastatin in experimental models significantly inhibited oxidative stress and the levels of inflammation-related proteins, [NLRP3, caspase-1, interleukin-1β (IL-1β), Toll-like receptor 4 (TLR4)], decreased pro-apoptotic-related proteins (cleaved caspase-3 and BAX), and increased anti-apoptotic proteins (Bcl-2) [117]. Also fenofibrate, which is a peroxisome proliferator-activated receptor α (PPARα) agonist, prevented heart damage in the wild-type 1 diabetic mice by reducing inflammation and fibrosis in cardiomyocytes [118]. 

The effects of lipid-lowering therapy in the clinical setting are still unknown. However, the use of statins is associated with an important decrease of cardiovascular risk in diabetic patients also in primary prevention [119]. Indeed, international guidelines suggest statins together with other lipid-lowering agents (PPARα agonists) for both primary and secondary CV prevention [120].

## 7. Conclusions

Although DC is rarely clinically evident, particularly in its early phase, it is a common condition, which increases the risk of the development of HF in people with diabetes. Its pathogenesis is still not completely clarified, although hyperglycemia, cardiac insulin resistance, and inflammatory pathways are key factors for myocardial fibrosis, stiffness, and hypertrophy. Achieving optimal glycemic control is important to prevent and delay diabetes complications and preserve heart health. Changes in lifestyle favoring healthy dietary patterns and physical activity, combined with more innovative anti-diabetes therapies are the current treatment strategies to safeguard the cardiovascular system. Data from randomized controlled trials show that both SGLT-2i and GLP-1RA offer protection against major cardiovascular events, including CV mortality and hospitalization for HF, in people with DMT2 at high CV risk. Future and larger studies are needed to better clarify the pathogenic mechanisms of pump dysfunction in diabetes and the effects of innovative therapies on DC and systolic and diastolic dysfunction.

## Figures and Tables

**Table 1:** Stages or subgroups of diabetic cardiomyopathy.

Characteristics	Stage 1	Stage 2	Stage 3	Stage 4
Progression	Early phase	Middle phase	Middle/late phase	Late phase
Function	Diastolic dysfunction	Diastolic and systolic dysfunction	Diastolic and systolic dysfunction	Diastolic and systolic dysfunction
Anatomy	Hypertrophy; ↑ LV mass	Hypertrophy; ↑ LV mass and wall thickness; dilatation; fibrosis	Dilatation; fibrosis; microangiopathy	Dilatation; fibrosis; micro- and macroangiopathy
Symptoms of HF	NYHA I	NYHA II	NYHA II-III	NYHA II-IV
Troponins	-	-	+ if inflammation or ischemia	+ in infarction or severe heart failure

↑ stands for increase; + stands for increased levels; HF, hearth failure; LV, left ventricle; NYHA, New York Heart Association.

**Table 2:** Summary of the main effects of different glucose-lowering agents on diabetic cardiomyopathy and heart failure.

Glucose-Lowering Agent	Mechanisms of Action	Effects on Pump Function
Metformin	↓ insulin resistance and TNF-α production ↓ cardiomyocytes and fibroblast LV remodeling ↑ production of NO ↑ systolic and diastolic function	No significant effects on HF hospitalization
SGLT-2i	↓ weight and blood pressure ↑ osmotic diuresis and natriuresis ↓ sodium-hydrogen exchanger (NHE) ↓ myocardial injury ↑ LV function	33% reduced risk of HF hospitalization
GLP-1RAs	↓ inflammatory myocardial remodeling ↓ inflammatory pathways in cardiomyocytes ↑ glucose uptake and coronary blood flow	10% reduced risk of HF hospitalization
DPP-4i	=/↑ diastolic function	No significant effect on HF hospitalization (↑ risk of HF hospitalization only with saxagliptin)
Sulfonylureas	↑ hypoglycemia risk ↑ weight	No significant effect on HF hospitalization
Thiazolidinediones	↑ weight ↑ edema ↓ inflammation, lipid and protein metabolism ↑ vascular endothelial function	↑ risk of HF hospitalization

↑ stands for increase;↓ stands for reduction; = stands for no effect. DPP-4i, dipeptidyl peptidase-4 inhibitor; GLP-1Ras, glucagon-like peptide-1 receptor agonist; HF, heart failure; SGLT-2i, Sodium-glucose cotransporter 2 inhibitors.
